# A-FABP Concentration Is More Strongly Associated with Cardiometabolic Risk Factors and the Occurrence of Metabolic Syndrome in Premenopausal Than in Postmenopausal Middle-Aged Women

**DOI:** 10.1155/2014/645762

**Published:** 2014-05-29

**Authors:** Anna Stefanska, Irena Ponikowska, Grazyna Sypniewska

**Affiliations:** ^1^Department of Laboratory Medicine, Collegium Medicum, Nicholas Copernicus University, Sklodowskiej-Curie 9, 85-094 Bydgoszcz, Poland; ^2^Department of Balneology, Collegium Medicum, Nicholas Copernicus University, Leśna 3, 87-720 Ciechocinek, Poland

## Abstract

We aimed at the evaluation of the relationship between adipocyte fatty acid binding protein (A-FABP) and cardiometabolic risk factors in premenopausal and postmenopausal women. Additionally, we compared A-FABP with adipokines related to metabolic syndrome (MetS) such as leptin and adiponectin. 94 premenopausal and 90 early postmenopausal middle-aged Caucasian women were subject to examinations. Postmenopausal women had higher A-FABP than premenopausal; this difference became insignificant after controlling for age. We found significantly higher correlation coefficients between A-FABP and TC/HDL-C ratio and number of MetS components in premenopausal women, compared to postmenopausal. Each 1 ng/dL increase in A-FABP concentration significantly increased the probability of occurrence of atherogenic lipid profile in premenopausal women, even after multivariate adjustment. All odds ratios became insignificant after controlling for BMI in postmenopausal women. A-FABP was more strongly associated with MetS than leptin and adiponectin in premenopausal women. Adiponectin concentration was a better biomarker for MetS after menopause. Our results suggest that the A-FABP is more strongly associated with some cardiometabolic risk factors in premenopausal than in postmenopausal women. Higher values of A-FABP after menopause are mainly explained by the fact that postmenopausal women are older. Because of the limitation of study, these results should be interpreted with caution.

## 1. Introduction

Adipose tissue secretes various adipokines, which are associated with cardiometabolic risk factors. In recent years, most attention has been given to the role of leptin and adiponectin in developing cardiometabolic events. Adipocyte fatty acid binding protein (A-FABP, FABP4) is an adipokine, which is a member of the fatty acid binding protein super family and is highly expressed in the adipose tissue [1]. In addition to production in adipocytes, this adipokine is also produced in significant amounts in macrophages and endothelial cells [2, 3]. A-FABP has been recently identified as a circulating biomarker of metabolic syndrome (MetS), type 2 diabetes, and cardiovascular events [4–6]. Both clinical investigations and animal studies identify A-FABP as a central mediator of obesity-related cardiovascular disease, possibly potentiating lipids-induced inflammation [1, 7]. In this study, we aimed at the evaluation of the relationship between A-FABP and the probability of the occurrence of cardiometabolic risk factors and metabolic syndrome in premenopausal and postmenopausal women. Although several studies have assessed the relationship between A-FABP and cardiometabolic risk factors in women, to our knowledge, no studies exist that compare premenopausal and postmenopausal women, whereas several studies have shown that A-FABP was more strongly related to cardiovascular events in women than in men [6, 8, 9].

## 2. Materials and Methods 

### 2.1. Participants

The study included 94 premenopausal middle-aged Caucasian women (40–51 years) who had regular, without changes in length, menstrual cycles in the past 12 months and had an early follicular phase FSH level <14.9 mIU/mL (late reproductive stage) and 90 early postmenopausal Caucasian women (46–60 years) who were from 1 to 8 years after the final menstrual period [10]. Women were recruited on their first day of sanatorium treatment at the Department of Balneology or selected from the group of women who participated in the metabolic syndrome study at the Department of Laboratory Medicine of the Nicholas Copernicus University in Bydgoszcz in the years from 2007 to 2010. Exclusion criteria were the following: thyroid or liver disease, diabetes mellitus type 1, chronic inflammation, cardiovascular diseases (CAD), surgical menopause, premature menopause, history of PCOS, thyroid treatment, insulin or oral antidiabetic therapy, hormonal replacement and lipid-lowering therapy, C-reactive protein (CRP) over 10 mg/L, and thyroid stimulating hormone (TSH) over 4.94 or less than 0.35 *μ*IU/mL. Clinical measurements (height, weight, waist circumference (WC), systolic blood pressure (SBP), and diastolic blood pressure (DBP)) were performed.

Women with any three of the following five criteria were considered to have metabolic syndrome: WC ≥ 80, glucose ≥ 5.6 mmol/L or previously diagnosed type 2 diabetes, triglycerides (TG) ≥ 1.7 mmol/L, HDL-cholesterol (HDL-C) < 1.29 mmol/L, and systolic pressure ≥130 or diastolic pressure ≥85 mmHg or the treatment of previously diagnosed hypertension) [11]. The homeostasis model assessment of insulin resistance (HOMA-IR) is defined by fasting insulin (mU/L) × glucose (mmol/L)/22.5. Ten-year risk for atherosclerotic cardiovascular disease (ASCVD) was calculated using the American Heart Association and the American College of Cardiology CV risk calculator. A level of 7.5% for 10-year ASCVD risk was considered as elevated [12]. Overweight and obesity were defined as BMI ≥ 25 kg/m^2^ and BMI ≥ 30 kg/m^2^, respectively. Elevated blood pressure (BP) was diagnosed if SBP was ≥130 mmHg and/or DBP was ≥85 mmHg. We accepted the following cutoff values: TC:HDL-C ≥ 4 and HDL-C < 50.0 mg/dL [13]; TG:HDL-C ≥ 1.3 [14] and CRP ≥ 1 mg/L [15]. Insulin resistance (IR) was defined as the HOMA-IR ≥ 3.4 (the third quartile for the Polish population) [16].

### 2.2. Biochemical Measurement

Fasting blood samples were collected. Premenopausal women were evaluated during the follicular phase (3–6 days of the menstrual cycle). Serum HDL-C, TG, TSH, total cholesterol (TC), LDL-cholesterol (LDL-C), free thyroxine (free T4), and plasma glucose were measured on the Architect ci8200 (Abbott Diagnostics). CRP was assayed by a high-sensitivity method (BN II, Dade Behring). Serum follicle-stimulating hormone (FSH) and 17*β*-estradiol (E2) were assayed on the AxSYM (Abbott Diagnostics) and on the Elecsys 1010/2010 (ROCHE Diagnostics), respectively. AFABP, insulin, adiponectin, sex hormone binding globulin (SHBG), and dehydroepiandrosterone sulphate (DHEAS) concentrations were assayed by ELISA (human A-FABP ELISA: Biovendor cat. number RD191036200R, CV intra-assay 3.9–6.6%, CV interassay 2.6–5.1%; insulin: DRG MedTek, intra-assay precision 2.8%–4.0%, interassay precision 2.6%–3.6%; human total adiponectin/Acrp30 R&D Systems, intra-assay precision 2.5%–4.7%, interassay 5.8%–6.9%; human leptin R&D systems, intra-assay precision 3.0%–3.3%, interassay 3.5%–5.4%; SHBG DRG MedTek, intra-assay precision 3.0%–8.6%, interassay 7.2%–11.6%; and DHEAS: Biovendor, intra-assay precision 7.5%–11.5%, interassay 4.2%–15.3%). All variables were measured for all subjects.

The study was approved by the Collegium Medicum Ethics Committee at Nicholas Copernicus University. All participants gave their written informed consents.

### 2.3. Statistical Analysis

The data were expressed as means (±standard error SE; Gaussian distribution) or medians (95% confidence interval; non-Gaussian distribution). The variables' normality was tested by the Shapiro-Wilk test. The variables were further compared by means of the Mann-Whitney *U* test (non-Gausssian) or by Student's *t*-test (Gaussian). Significant differences between groups were also tested with the analysis of covariance (ANCOVA) with adjustment for age. The distributions of HOMA-IR, TG/HDL-C ratio, 10-year ASCVD risk, and levels of glucose, insulin, TG, CRP, FSH, E2, SHBG, and DHEAS were markedly skewed. Thus, these parameters were normalized by log transformation. Pearson and partial correlation coefficients were computed to assess the association between AFABP and various parameters. The difference between two correlation coefficients was computed using the Fisher* r*-to-*z* transformation. In the multiple linear regression analysis such variables as age, smoking status, BMI SBP, log HOMA-IR, log CRP, log TG, and TC/HDL-C ratio (PRE) or HDL-C (POST) were assayed as independent variables, whereas log A-FABP was assessed as a dependent variable. Logistic regression was applied. In all logistic models AFABP was included. Additional models were adjusted for age, years since menopause, BMI, HOMA-IR, smoking, physical activity, SBP, TG/HDL-C ratio, adiponectin, and leptin. The significance of AFABP coefficients in the logistic models was tested by the Wald chi-squared statistics. The goodness of fit of models was evaluated by Hosmer and Lemeshow chi-square test. Additionally, ROC curves for single laboratory parameters were constructed and the areas under the curve with 95% confidence interval were calculated (AUC, 95% CI; thresholds with sensitivity and specificity). The level of statistical significance is chosen as 0.05 (Statistica 9, StatSoft).

## 3. Results

The postmenopausal women had significantly higher age, A-FABP, SBP, DBP, glucose, TG, TC, LDL-C, TC/HDL-C ratio, FSH, leptin, and 10-year ASCVD risk and significantly lower E2 and DHEAS in comparison with premenopausal women. All statistically significant differences in means or medians between groups (except TC, E2, and FSH) became statistically insignificant after controlling for age (A-FABP *P* = 0.07; SBP *P* = 0.5; DBP *P* = 0.53; glucose *P* = 0.44; LDL-C *P* = 0.20; TC/HDL-C ratio *P* = 0.30; leptin *P* = 0.28; and 10-year ASCVD risk *P* = 0.29). The prevalence of MetS, MetS components (except WC and elevated HDL-C), and 10-year ASCVD risk was higher in postmenopausal women. The premenopausal and postmenopausal women were matched for BMI, WC, and prevalence of overweight, obesity, central obesity, smoking, and low physical activity (Table 1). The concentration of A-FABP was statistically significantly higher in all cardiometabolic disturbances in premenopausal women (Table 2).  Table 3 shows unadjusted Pearson correlations. In premenopausal women AFABP was significantly correlated with all parameters listed except TC and FSH. After being adjusted for age and BMI, A-FABP was significantly correlated with glucose (partial *r* = 0.21,  *P* = 0.04), HOMA-IR (partial *r* = 0.22,  *P* = 0.03), HDL-C (partial *r* = −0.21, *P* = 0.04), TC/HDL-C (partial *r* = 0.43, *P* < 0.001), TG/HDL-C (partial *r* = 0.23, *P* = 0.03), CRP (partial *r* = 0.22,  *P* = 0.04), E2 (partial *r* = −0.21, *P* = 0.04), number of MetS components (partial *r* = 0.24, *P* = 0.02), and 10-year ASCVD risk (partial *r* = 0.25, *P* = 0.01). After menopause only negative correlation between A-FABP and HDL-C remained significant after controlling for age and BMI (partial *r* = −0.28, *P* = 0.01). We observed that the values of the unadjusted Pearson correlation coefficients calculated for age, glucose, insulin, HOMA-IR, LDL-C, TC/HDL-C ratio, TG/HDL-C ratio, E2, SBP, DBP, number of MetS components, and 10-year ASCVD risk were higher in premenopausal women in comparison to postmenopausal women, but a statistically significant difference between two correlation coefficients was found only for TC/HDL-C ratio and number of MetS components (Table 3). Multiple linear regression analysis performed with log-transformed A-FABP as the dependent variable and with age, smoking status, BMI SBP, log HOMA-IR, log CRP, log TG, and TC/HDL-C ratio (PRE) or HDL-C (POST) as the continuous independent variables showed significant and independent associations of A-FABP levels with BMI and TC/HDL-C ratio (*R* ^2^ = 0.58; *β* = 0.48, *P* < 0.001; *β* = 0.45, *P* < 0.001, resp.) in premenopausal women and significant and independent associations of A-FABP levels with BMI and HDL-C (*R* ^2^ = 0.32; *β* = 0.40, *P* = 0.002; *β* = −0.37, *P* = 0.008, resp.) in postmenopausal women. BMI accounted for 37% of A-FABP variability and TC/HDL-C ratio accounted for 16% of A-FABP variability in premenopausal women. BMI accounted for 22% of A-FABP variability and HDL-C accounted for 8.2% of A-FABP variability in postmenopausal women (data not shown in tables).

In premenopausal women, the logistic regression analysis (Wald test) presented significant associations of A-FABP with the probability of MetS and all cardiometabolic risk factors in the unadjusted model as well as in all models adjusted for age or years since menopause (except elevated glucose and TG). The association of A-FABP with the probability of MetS, insulin resistance, and elevated blood pressure became statistically insignificant, after including BMI in the regression model. The association of A-FABP with the probability of elevated CRP became statistically insignificant after adjustment for additional potential confounding factors such as smoking, physical activity, HOMA-IR, SBP, and TG/HDL-C ratio. In the unadjusted model, each 1 ng/dL increase in A-FABP increased the probability of MetS by 5.7%, HOMA-IR ≥ 3.4 by 3.7%, hypertension by 3.5%, HDL-C < 50 mg/dL by 17%, TC/HDL-C ≥ 4 by 8.3%, TG/HDL-C ≥ 1.3 by 4%, CRP ≥ 1.0 by 9.1%, and obesity by 18%.

The lowest values of ORs were found in postmenopausal women. After being adjusted for BMI or for other potential confounding factors, all values of OR became statistically insignificant (Table 4). The unadjusted model with 10-year ASCVD risk was statistically insignificant in postmenopausal women. It was not possible to design any model for 10-year ASCVD risk in premenopausal women because no women had risk ≥7.5%.

We also constructed ROC curves to assess diagnostic accuracies of A-FABP for the prediction of the occurrence of cardiometabolic risk factors. The statistically significant higher levels of discrimination of Mets, HOMA-IR ≥ 3.4, TC/HDL-C ≥ 4, and hypertension were found for A-FABP in premenopausal women in comparison with postmenopausal women.

For all cardiometabolic risk factors, sensitivity and specificity were calculated for two different cutoff points (Table 5). The ROC curve constructed for 10-year ASCVD risk showed that A-FABP had poor discriminating power for predicting 10-year ASCVD risk in postmenopausal women (AUC 0.66 (0.48–0.79)).

Finally, we compared A-FABP with adipokines related to MetS such as leptin and adiponectin. We included adiponectin and leptin in logistic regression models adjusted for age. We observed that A-FABP and adiponectin were significantly associated with the probability of MetS occurrence (A-FABP OR per unit 1.062 (1.01–1.11), *P* = 0.01; adiponectin OR per unit 0.86 (0.74–1.0), *P* = 0.044; and leptin OR per unit 0.98 (0.92–1.032), *P* = 0.39) in premenopausal women. In postmenopausal women, adiponectin was the only one significantly associated with the probability of MetS occurrence (adiponectin OR per unit 0.85 (0.77–0.98), *P* = 0.02; leptin OR per unit 1.046 (0.99–1.11), *P* = 0.11; and A-FABP OR per unit 0.97 (0.94–1.0), *P* = 0.23). Adiponectin and leptin had lower values of AUC for MetS in comparison with A-FABP in premenopausal women (adiponectin AUC = 0.75 (0.62–0.88), *P* = 0.05; leptin AUC = 0.61 (0.48–0.74); and A-FABP AUC = 0.90 (0.84–0.96)), whereas adiponectin and leptin had higher values of AUC for MetS in comparison with A-FABP in postmenopausal women [adiponectin AUC = 0.72 (0.60–0.85); leptin AUC = 0.68 (0.55–0.81); and A-FABP AUC = 0.56 (0.43–0.69)] (data not shown in tables).

## 4. Discussion 

In this case-control study, we separately evaluated—for the first time—the A-FABP in premenopausal and postmenopausal women. Previous studies have found that A-FABP concentrations had a significant association with cardiometabolic risk factors, MetS, and cardiovascular diseases in women with their average age between 40 and 60 years [5, 6, 9, 17–23]. We studied the group of middle-aged women because this group is especially exposed to the increased risk of developing the cardiometabolic profile. Despite the fact that premenopausal and postmenopausal women were middle-aged, we found that postmenopausal women were significantly older; the difference in age between groups was 10 years on average. In the unadjusted analysis, A-FABP concentration was significantly higher in postmenopausal women than in premenopausal women. After controlling for age, the difference in the A-FABP concentration between premenopausal and postmenopausal women became statistically insignificant. A-FABP concentrations are strongly correlated with BMI and WC because the adipose tissue is a major source of circulating A-FABP [1]. However, in this study these indicators of body mass did not significantly affect the difference in the A-FABP concentration because both groups were matched for BMI, WC, and the prevalence of overweight and obesity. Therefore, we may conclude that the significantly higher values of A-FABP may be partially explained by the fact that postmenopausal women were older, which is consistent with the fact that A-FABP concentrations grow with age [20, 21]. We also observed that postmenopausal women had significantly higher values of most common cardiometabolic risk factors and higher prevalence of MetS and 10-year ASCVD risk than premenopausal women, which is consistent with other authors [24, 25]. Questions remain whether menopause has a causative contribution to the deteriorating metabolic profile that is independent of chronological aging. We observed that after controlling for age, the differences for most cardiovascular risk factors became statistically insignificant, which may suggest the significant influence of aging on cardiometabolic risk profile after menopause. However, due to limitations of this study, we cannot exclude a direct effect of menopause on an increased cardiometabolic risk profile [26].

Statistical results of this study suggest that A-FABP is more strongly associated with some cardiometabolic risk factors in premenopausal than in postmenopausal women. We observed that the values of correlation coefficients calculated for most of the cardiometabolic risk factors were higher in premenopausal women, but a statistically significant difference between two correlation coefficients was found only for TC/HDL-C ratio and a number of MetS components. Moreover, we observed that all of significant correlations (except hypertension) persisted after adjusting for age and BMI, which may suggest that A-FABP activity is additionally controlled by other factors than age and body mass in premenopausal women. After menopause, only a negative correlation between A-FABP and HDL-C remained significant after controlling for age and BMI. However, it seems that HDL-C is not affected significantly by A-FABP because we observed higher values of HDL-C after menopause, which is probably associated with changes in the lipoprotein subclass profile observed during the menopausal transition [27]. Additionally, the results of our logistic regression analysis showed that each 1 ng/dL increase in the A-FABP concentration significantly increased the probability of the occurrence of obesity, elevated TC/HDL-C ratio and TG/HDL-C ratio, and decreased HDL-C level even after multivariate adjustment, whereas all odds ratios became statistically insignificant after controlling for BMI in postmenopausal women. On the other hand, the higher values of A-FABP in postmenopausal women, which are more strongly affected by cardiometabolic risk factors, may suggest that this adipokine had strong association with an increased cardiometabolic risk profile after menopause. However, our statistical analysis suggests that significantly higher concentrations of A-FABP and values of the cardiometabolic risk factors were mainly affected by the higher age of postmenopausal women. Therefore, for better statistical analysis, we should enroll premenopausal and postmenopausal women with comparable values of age, which is very difficult to perform. Most of the previous studies have estimated the concentration of A-FABP in different groups, which were matched for age, which is very important due to the fact that the A-FABP level is growing with age [20, 21].

Only one study has recently assessed the A-FABP concentration in apparently healthy premenopausal women, without contraception. The authors of this paper have found—comparable to our values—correlation coefficients between A-FABP and cardiometabolic risk factors [28]. Thus, we can speculate that the A-FABP concentration is similarly associated with cardiometabolic risk factors in young and middle-aged premenopausal women. Moreover, only one study evaluated the serum A-FABP in the group, where all women were postmenopausal. The authors of this paper have found that the A-FABP concentration is independently associated with HOMA-IR and LDL-C, which is contrary to our results [29]. Yet, in the cited paper, all women had high risk of CAD and underwent coronary angiography. Our postmenopausal women had no history of cardiovascular events and only 9.4% had a slightly increased 10-year ASCVD risk. Therefore, it seems that future studies are necessary to evaluate the relationship between A-FABP and cardiometabolic risk factors in postmenopausal women with and without a high risk of CAD.

We used the ROC curves to compare the discriminatory power of A-FABP in premenopausal and postmenopausal women. We found that A-FABP had statistically significantly higher levels of discrimination of MetS, hypertension, IR, and TC/HDL-C ≥ 4 in premenopausal women than in postmenopausal women. However, these results should be interpreted with caution because of the small sample size, and future studies with a larger sample size would be beneficial to evaluate clinical usefulness of A-FABP in premenopausal and postmenopausal women.

Finally, we compared A-FABP with other adipokines related to MetS such as leptin and adiponectin in premenopausal and postmenopausal women. Our results of the logistic regression analysis and ROC curves suggest that the A-FABP concentration assesses the probability of MetS similarly to or even better than adiponectin and better than leptin in premenopausal women, whereas the adiponectin concentration seems to be a better biomarker for MetS than A-FABP and leptin in postmenopausal women. Henneman et al. have also observed that menopause reinforces the relationship between adiponectin and indicators of MetS [30].

The stronger association of A-FABP with cardiometabolic risk factors in premenopausal women may be caused by clinical differences between premenopausal and postmenopausal women or may be associated with a statistical phenomenon. For that reason, our findings should be interpreted with caution. The postmenopausal women had higher values of cardiometabolic risk factors and A-FABP along with the narrower ranges of these parameters, which could hinder the associations between variables. Therefore, in order to assess the impact of menopause on A-FABP concentration more effectively, we should enroll premenopausal and postmenopausal women with comparable values of cardiometabolic risk factors.

On the other hand, the stronger association of A-FABP with cardiometabolic risk factors before menopause may be partly the result of the menopause fat redistribution, as premenopausal women generally have more subcutaneous fat than postmenopausal women, whereas postmenopausal women have more visceral fat. Toth et al. have suggested that the early postmenopausal status is associated with a preferential increase in intra-abdominal fat that is independent of age and the total body fat mass [31]. The menopause fat redistribution may affect the concentration of A-FABP because the production of this adipokine is substantially higher in subcutaneous fat compared with visceral fat, and recently published studies have clearly indicated that A-FABP is not the mediator of the visceral fat effect [32]. For that reason we could formulate a hypothesis that the A-FABP concentration could be lower or activity of this adipokine could be weaker after menopause. In this study, WC values did not differ between premenopausal and postmenopausal women. However, Kuk et al. have clearly shown that the amount of visceral fat associated with a given WC is markedly affected by the age of the individual. For example, an older woman (50 years of age) with a WC of 102 cm would be expected to have 70% more visceral fat than would a 25-year-old woman with the same WC and 43% more visceral fat than would a 40-year-old woman with the same WC. Thus, it is clear that differences in visceral fat and the abdominal subcutaneous fat distribution may not be reflected by differences in its surrogate measure, that is, WC [33, 34]. In this study we had no possibilityofmeasuring the visceral and subcutaneous adipose tissue areas; therefore, future studies need to be performed to assess whether the body fat redistribution is associated with the A-FABP concentration after menopause. It has only been observed that fatty acid metabolites increase in visceral fat (but not in subcutaneous fat after menopause), which might be related to metabolic syndrome in postmenopausal women [35]. Previous studies have also observed a lower concentration of A-FABP in men and a weaker association of this adipokine with cardiometabolic risk factors, atherosclerosis, and coronary artery disease in men than in women, which is mainly explained by sexual dimorphism in fat distribution, as women generally have more subcutaneous fat than men [6, 8, 9]. On the other hand, cardiometabolic risk factors are more strongly associated with visceral fat, which can outweigh the impact of A-FABP on the cardiometabolic status, and this may result in a weaker association of this adipokine with risk factors after menopause [36, 37].

Another possible explanation for the stronger association of A-FABP with cardiometabolic risk factors prior to menopause is the impact of sex hormones. In our study we observed that the A-FABP concentration was negatively correlated with E2 and this correlation was independent of age and BMI in premenopausal women. This correlation was weak but may suggest that higher values of estradiol decrease the concentration of A-FABP, and this may partially explain the lower values of A-FABP in premenopausal women. Additionally, we found that markers of androgenization (SHBG and DHEAS) were not independently associated with A-FABP before and after menopause, whereas a significant correlation between A-FABP and FSH was mainly explained by values of BMI in postmenopausal women. There is only one study reporting theassociation of A-FABP with markers of hyperandrogenism, in which the authors did not find an independent relationship between A-FABP and testosterone, free testosterone, SHBG, and DHEAS in the polycystic ovary syndrome women [38]. On the other hand, Yeung et al. have observed that the sexual dimorphism persisted among subjects over the age of 55, which may suggest that estrogen might not be important in regulating the A-FABP production in women, but this hypothesis seems to be insufficient due to the fact that the group has not been divided into women before and after menopause [9].

## 5. Conclusion 

Our statistical results suggest that the A-FABP concentration is more strongly associated with some cardiometabolic risk factors (especially lipid risk factors) in premenopausal than in postmenopausal women, whereas higher values of A-FABP after menopause are mainly explained by the fact that postmenopausal women are older. However, because of the limitation of this study, these results should be interpreted with caution and future studies need to be performed to assess whether A-FABP could be used as an equivalent biomarker for cardiometabolic disturbances in premenopausal and postmenopausal women.

The present study has certain limitations. The study sample was not randomly recruited, which means that our findings may not be applicable to the general population of middle-aged women. Premenopausal and postmenopausal women were not matched by age and cardiometabolic risk, which may hinder statistical analysis of A-FABP. Another limitation of our study was the relatively small sample size. In this study the prevalence of obesity was nearly 50% in premenopausal and postmenopausal women. For that reason it seems that A-FABP should be also evaluated in the groups of women with a lower prevalence of obesity. Another limitation is that this study does not include perimenopausal women. We analyzed premenopausal and postmenopausal women because we believe that these groups more explicitly show the effect of menopause on the A-FABP concentration.

## Figures and Tables

**Table 1:** Characteristics of the premenopausal and postmenopausal women.

Parameters	PRE *n* = 94	POST *n* = 90	*P* PRE versus POST
Age [years]	44.3 ± 0.4	54.2 ± 0.4	<0.001
BMI [kg/m^2^]	29.0 ± 0.7	30.1 ± 0.7	0.21
WC [cm]	90.4 ± 1.7	92.8 ± 1.5	0.23
A-FABP [ng/mL]	21.4 (14.2–28.4)	31.6 (23.6–47.9)	<0.001^#^
SBP [mmHg]	121.8 ± 1.6	130.9 ± 2.3	<0.001^#^
DBP [mmHg]	78.0 ± 1.0	83.9 ± 1.3	0.001^#^
Glucose [mmol/L]	4.99 (4.61–5.38)	5.44 (5.11–5.88)	<0.001^#^
HDL-C [mmol/L]	1.49 ± 0.03	1.58 ± 0.04	0.07
TG [mmol/L]	1.11 (0.92–1.25)	1.21 (0.93–1.65)	0.01^#^
TC [mmol/L]	5.1 ± 0.1	5.8 ± 0.1	<0.001
LDL [mmol/L]	3.1 ± 0.1	3.6 ± 0.1	<0.001^#^
TC/HDL-C ratio	3.5 ± 0.1	3.8 ± 0.1	0.015^#^
TG/HDL-C ratio	0.78 (0.51–0.97)	0.86 (0.57–1.15)	0.14
CRP [mg/L]	1.13 (0.60–2.68)	1.42 (0.71–3.34)	0.17
Adiponectin [*µ*g/mL]	7.8 (5.2–12.2)	9.9 (6.1–14.9)	0.08
Leptin [ng/mL]	21.2 (13.8–35.6)	29.9 (17.7–37.9)	0.04
Insulin [mU/L]	6.2 (3.6–8.8)	6.2 (4.0–9.4)	0.45
HOMA-IR	1.44 (0.62–1.97)	1.42 (1.03–2.48)	0.28
FSH [mIU/mL]	6.9 (5.4–9.2)	65.5 (52.1–85.6)	<0.001
E2 [pg/mL]	52.0 (39.9–81.0)	19.0 (11.0–29.0)	<0.001
SHBG [nmolL]	41.4 (30.7–54.8)	37.5 (25.2–53.4)	0.18
DHEAS [*µ*g/mL]	1.80 (1.21–2.26)	1.47 (1.0–1.97)	0.05
TSH [*µ*IU/mL]	1.57 (0.99–2.67)	1.31 (0.83–2.14)	0.14
fT4 [ng/dL]	1.0 ± 0.02	1.03 ± 0.02	0.1
10-year ASCVD risk [%]	0.8 (0.4–1.3)	2.5 (1.7–4.3)	<0.001^#^
10-year ASCVD risk ≥7.5%	0%	9.4%	0.002
MetS	19%	43%	0.004
Elevated BP^!^	31%	56%	<0.001
Glucose ≥ 5.6 mmol/L	19%	39%	0.003
HDL < 1.29 mmol/L	31%	17%	0.03
TG ≥ 1.7 mmol/L	13%	24%	0.05
WC ≥ 80 cm	82%	88%	0.26
BMI 18.5–24.9	25%	17%	0.18
BMI 25–29.9	30%	34%	0.56
BMI ≥ 30	45%	49%	0.59
Current smokers	19%	23%	0.5
Alcohol consumption: never or occasionally	83%	94%	0.02
Physical activity: never or sporadically	42%	51%	0.22

Means (±SE) or medians (95% confidence interval); mean (min–max) or % (*n*) (&); ns: not statistically significant; final menstrual period (FMP); *P*: probability of error for testing equal distributions in groups. Blood pressure (BP), ^!^elevated BP (≥130/≥85 mmHg), or hypertension treatment. ^#^Statistically insignificant after controlling for age (ANCOVA).

**Table 2:** Concentration of A-FABP in cardiometabolic disturbances.

	PRE	POST
MetS (−)	19.5 (13.5–23.8)	28.9 (21.5–48.4)
MetS (+)	35.5 (19.6–41.7)^#^	36.3 (26.9–47.0)
HOMA < 3.4	20.2 (13.5–25.8)	29.8 (23.4–47.9)
HOMA ≥ 3.4	36.0 (29.7–36.9)^#^	37.4 (29.5–58.7)
Elevated BP (−)^!^	20.2 (13.0–24.9)	30.9 (24.1–52.0)
Elevated BP (+)	29.6 (23.2–36.0)^#^	34.0 (24.1–48.1)
HDL-C ≥ 50 mg/dL	17.6 (13.0–23.0)	29.1 (22.6–41.7)
HDL-C < 50 mg/dL	35.5 (21.9–41.7)^#^	48.1 (28.9–76.2)^@^
TC/HDL-C < 4	18.3 (13.0–23.8)	29.1 (23.4–47.9)
TC/HDL-C ≥ 4	31.4 (23.2–36.8)^#^	32.8 (25.3–48.1)
TG/HDL-C < 1.3	20.1 (13.3–25.3)	30.1 (22.6–47.9)
TG/HDL-C ≥ 1.3	28.4 (22.5–33.7)^@^	38.6 (28.9–59.3)
CRP mg/L < 1.0	16.2 (12.9–21.5)	24.9 (18.4–39.9)
CRP mg/L ≥ 1.0	23.2 (18.3–34.3)^#^	36.9 (27.8–54.5)^@^
BMI < 30	17.3 (13.0–21.3)	25.8 (20.5–32.6)
BMI ≥ 30	31.4 (22.8–36.9)^#^	46.0 (32.1–59.3)^#^

*P* < 0.05; ^@^ *P* < 0.01; ^#^ *P* < 0.001.

^!^Elevated BP (≥130/≥85 mmHg) or hypertension treatment.

**Table 3:** Pearson correlation coefficients between log A-FABP and measured parameters in pre- and postmenopausal women.

	Log A-FABP PRE	Log A-FABP POST	*P* ^a^ PRE versus POST
Age	0.21*	0.08	0.37
BMI	0.63^#^	0.56^#^	0.46
WC	0.53^#^	0.47^#^	0.59
log glucose	0.27*	0.12	0.29
log insulin	0.42^#^	0.27*	0.25
log HOMA-IR	0.44^#^	0.29^!^	0.24
TC	0.15	0.11	0.78
LDL-C	0.20*	−0.08	0.06
HDL-C	−0.43^#^	−0.34^!^	0.48
TC/HDL-C	0.57^#^	0.19	0.003
log TG	0.35^#^	0.23^!^	0.38
log TG/HDL-C	0.45^#^	0.29^!^	0.21
log CRP	0.45^#^	0.36^#^	0.47
log FSH	−0.02	−0.27*	0.08
log E2	−0.25*	−0.07	0.21
SBP	0.31^!^	0.04	0.06
DBP	0.25*	0.02	0.011
log adiponectin	−0.30^!^	−0.28^!^	0.88
log DHEAS	0.09	−0.18	0.54
log SHBG	−0.32^#^	−0.26*	0.66
Number of MetS components	0.54^#^	0.29^!^	0.04
log 10-year ASCVD risk	0.39^#^	0.18	0.12

*P < 0.05; ^!^ *P* < 0.01; ^#^ *P* < 0.001; premenopausal women (PRE); postmenopausal women (POST). Comparison of correlation coefficients PRE versus POST (*P* ^a^).

**Table 4:** Logistic regression models for the unadjusted and adjusted associations of A-FABP with MetS, MetS components, and cardiometabolic risk factors.

	Unadjusted OR per unit (95% CI)		OR per unit adjusted for age and BMI^$^		Multivariate-adjusted OR per unit (95% CI)^&^	
	PRE	POST	PRE	POST	PRE	POST
MetS	1.06^@^ (1.01–1.10)	1.0 (0.98–1.02)	1.02 (0.98–1.06)	0.97 (0.95–1.01)	1.02 (0.97–1.04)	0.91 (0.79–1.06)
Glucose ≥ 5.6 mmol/L	1.0 (0.97–1.03)	0.99 (0.97–1.02)	0.99 (0.94–1.03)	0.98 (0.97–1.01)	0.99 (0.94–1.03)	0.99 (0.95–1.02)
HDL-C < 1.29 mmol/L	1.17^#^ (1.09–1.25)	1.03* (1.01–1.06)	1.13^#^ (1.04–1.24)	1.02 (0.99–1.04)	1.08^#^ (1.03–1.18)	1.02 (0.99–1.05)
TG ≥ 1.7 mmol/L	1.0 (0.97–1.04)	1.0 (0.97–1.03)	1.0 (0.95–1.05)	1.0 (0.97–1.03)	0.94 (0.92–1.07)	1.01 (0.97–1.04)
WC ≥ 80 cm	1.20^#^ (1.08–1.33)	1.09^#^ (1.05–1.22)	1.23^#^ (1.09–1.40)	1.09^#^ (1.05–1.24)	1.24^#^ (1.09–1.61)	1.07^#^ (1.03–1.27)
Elevated BP (≥130/≥85 mmHg)^!^	1.04* (1.01–1.07)	1.0 (0.99–1.01)	0.99 (0.96–1.03)	0.98 (0.96–1.02)	0.98 (0.95–1.02)	0.98 (0.96–1.02)
HOMA ≥ 3.4	1.04* (1.01–1.07)	1.01 (0.98–1.04)	1.01 (0.97–1.06)	0.98 (0.94–1.03)	0.99 (0.95–1.02)	0.97 (0.90–1.04)
TC/HDL-C ≥ 4	1.08^#^ (1.03–1.135)	1.0 (0.98–1.02)	1.14^#^ (1.05–1.25)	1.0 (0.98–1.03)	1.14^#^ (1.03–1.26)	1.01 (0.98–1.03)
TG/HDL-C ≥ 1.3	1.04^@^ (1.01–1.07)	1.01 (0.99–1.04)	1.06^@^ (1.0–1.11)	1.0 (0.98–1.04)	1.06^@^ (1.02–1.11)	1.02 (0.98–1.05)
CRP mg/L ≥ 1.0	1.09^#^ (1.03–1.15)	1.04* (1.01–1.07)	1.05^@^ (0.98–1.13)	1.01 (0.98–1.04)	0.96 (0.90–1.03)	1.0 (0.97–1.04)
BMI ≥ 30	1.18^#^ (1.10–1.27)	1.06^#^ (1.02–1.10)	1.18^#^ (1.09–1.27)	1.06^#^ (1.02–1.09)	1.15^#^ (1.06–1.24)	1.04* (1.0–1.07)

Odd ratio (OR); confidence interval (CI); premenopausal women (PRE); postmenopausal women (POST); metabolic syndrome (MetS); blood pressure (BP); ^!^elevated BP or hypertension treatment. According to chi-square statistic all models achieved statistical goodness of fit. Wald test significance: *P < 0.05; ^@^ *P* < 0.01; ^#^ *P* < 0.001.

All ORs remained statistically significant after adjustment for age. Similar results were obtained when age was replaced by years since menopause in postmenopausal women.

^$^Model adjusted for age and BMI (except BMI when modeling associations for elevated BMI and elevated WC). ^&^Multivariate logistic regression model adjusted for age, BMI, smoking, physical activity, HOMA-1, SBP, and TG/HDL-C ratio (except BMI when modeling associations for elevated BMI and elevated WC; except HOMA-IR when modeling associations for elevated glucose and elevated HOMA-IR; except SBP when modeling association for elevated BP; except TG/HDL-C ratio when modeling associations for elevated TG, decreased HDL-C, elevated TG/HDL-ratio, and elevated TC/HDL-C ratio).

**Table 5:** Diagnostic utility of A-FABP for predicting cardiometabolic risk factors and metabolic syndrome.

	PRE AUC (95% CI)	POST AUC (95% CI)	*P* PRE versus POST	Cutoff	PRE Sensitivity/specificity (%)	POST Sensitivity/specificity (%)
MetS	0.90 (0.84–0.96)	0.56 (0.43–0.69)	<0.001	27.0 30.0	88/87 78/87	75/40 62/52

HOMA-IR ≥ 3.4	0.85 (0.76–0.94)	0.61 (0.40–0.81)	0.04	27.0 30.0	80/79 80/81	88/36 75/49

HDL-C < 50	0.85 (0.75–0.95)	0.73 (0.57–0.89)	0.25	27.0 35.0	73/94 53/100	82/39 73/60

TC/HDL-C ≥ 4	0.84 (0.76–0.92)	0.53 (0.40–0.66)	<0.001	21.0 30.0	92/65 61/89	84/18 74/40

TG/HDL-C ≥ 1.3	0.75 (0.67–0.86)	0.64 (0.50–0.78)	0.22	21.0 30.0	88/55 50/80	100/21 71/51

CRP ≥ 1	0.74 (0.64–0.84)	0.71 (0.59–0.83)	0.73	21.0 30.0	68/67 36/91	91/31 66/69

Elevated BP (≥130/≥85 mmHg)^!^	0.76 (0.66–0.85)	0.52 (0.38–0.65)	0.03	27.0 35.0	67/82 44/90	69/31 49/56

BMI ≥ 30	0.83 (0.75–0.93)	0.79 (0.68–0.89)	0.51	21.0 30.0	86/70 52/96	78/72 91/31

Area under the curve (AUC), confidence interval (CI), blood pressure (BP), premenopausal women (PRE), and postmenopausal women (POST). ^!^Elevated BP or hypertension treatment.
